# Satellite Tracking of Sympatric Marine Megafauna Can Inform the Biological Basis for Species Co-Management

**DOI:** 10.1371/journal.pone.0098944

**Published:** 2014-06-03

**Authors:** Christian Gredzens, Helene Marsh, Mariana M. P. B. Fuentes, Colin J. Limpus, Takahiro Shimada, Mark Hamann

**Affiliations:** 1 School of Earth and Environmental Sciences, James Cook University, Townsville, Queensland, Australia; 2 School of Marine and Tropical Biology, James Cook University, Townsville, Queensland, Australia; 3 Centre for Tropical Water and Aquatic Ecosystem Research, James Cook University, Townsville, Queensland, Australia; 4 ARC Centre of Excellence for Coral Reef Studies, James Cook University, Townsville, Queensland, Australia; 5 Aquatic Threatened Species Unit, Queensland Department of Environment and Heritage Protection, Brisbane, Queensland, Australia; Deakin University, Australia

## Abstract

**Context:**

Systematic conservation planning is increasingly used to identify priority areas for protection in marine systems. However, ecosystem-based approaches typically use density estimates as surrogates for animal presence and spatial modeling to identify areas for protection and may not take into account daily or seasonal movements of animals. Additionally, sympatric and inter-related species are often managed separately, which may not be cost-effective. This study aims to demonstrate an evidence-based method to inform the biological basis for co-management of two sympatric species, dugongs and green sea turtles. This approach can then be used in conservation planning to delineate areas to maximize species protection.

**Methodology/Results:**

Fast-acquisition satellite telemetry was used to track eleven dugongs and ten green turtles at two geographically distinct foraging locations in Queensland, Australia to evaluate the inter- and intra-species spatial relationships and assess the efficacy of existing protection zones. Home-range analysis and bathymetric modeling were used to determine spatial use and compared with existing protection areas using GIS. Dugong and green turtle home-ranges significantly overlapped in both locations. However, both species used different core areas and differences existed between regions in depth zone use and home-range size, especially for dugongs. Both species used existing protection areas in Shoalwater Bay, but only a single tracked dugong used the existing protection area in Torres Strait.

**Conclusions/Significance:**

Fast-acquisition satellite telemetry can provide evidence-based information on individual animal movements to delineate relationships between dugongs and green turtles in regions where they co-occur. This information can be used to increase the efficacy of conservation planning and complement more broadly based survey information. These species also use similar habitats, making complimentary co-management possible, but important differences exist between locations making it essential to customize management. This methodology could be applied on a broader scale to include other sympatric and inter-related species.

## Introduction

Understanding how animals use space can be fundamental to identifying the components necessary for the conservation of a species [1]. These components may include key resources, critical habitats, movement patterns, and distributions; all of which can be used to determine the spatial dynamics of a population [2]–[4]. Such knowledge has the potential to play a key role in the designation of marine protected areas (MPAs), guiding management decisions, predicting the effects of environmental change, and aiding in the calculation of population estimates [5]–[7]. Knowledge of these components is especially important as the human population continues to grow and anthropogenic influences become more widespread throughout the natural environment [8].

MPAs are being increasingly used to protect marine species and environments [9], [10], especially as the number of marine species listed as threatened increases [11]. However, single species approaches to MPA design are the subject of increased scrutiny with ecosystem-based approaches being increasingly favored [12]–[15]. Contemporary ecosystem-based approaches depend on using acceptable surrogates for the presence of species or species groups in conjunction with sighting records, density estimates, and appropriate modeling techniques [9], [16]. All of these factors rely on assumptions that are vulnerable to errors of commission and omission as well as sampling bias which can affect MPA efficacy [16], [17]. These concerns are exacerbated when designing MPAs to conserve species that are mobile and/or dependent on benthic habitats of variable quality.

The use of MPAs to protect mobile marine species, such as marine megavertebrates (e.g., sea turtles, marine mammals, and elasmobranchs), can be difficult as their ranges may encompass significant portions of coastlines and even entire ocean basins [9], [18]–[20] (but see [21]). Additionally, a diverse range of cultural, economic, political, and legal obstacles can impede the establishment and enforcement of large reserves, especially transnational reserves [22], [23]. However, many mobile marine megavertebrates display strong site fidelity to specific regions on a seasonal or yearly basis allowing the protection of key areas using permanent or seasonal spatial closures [9], [20], [24]. This protection can be particularly important for marine megavertebrates as many species display life-history characteristics (e.g., long lived, low fecundity, high adult survivorship) [25]–[27] that increase their vulnerability to human-induced mortality and destruction of critical habitat.

Using a systematic conservation planning approach to designate high conservation value areas to conserve marine megavertebrates has increased over the past decade [10], [15], [20], [28]. This planning methodology was a key driver in the implementation of conservation areas for dugongs (*Dugong dugon*) and green sea turtles (*Chelonia mydas*) in the rezoning of the Great Barrier Reef Marine Park (GBRMP), Australia in 2003 and has been suggested for use in implementing reserves in Torres Strait, Australia [10], [29]. These regions both contain globally significant populations of dugongs and green sea turtles, with Torres Strait being home to the World's largest dugong population (>12,000 individuals) [26] while the Great Barrier Reef (GBR) and Torres Strait region contain some of the most important nesting grounds for the largest population of green sea turtles [30]. The rezoning of the GBR in 2003 increased the level of protection of high conservation value foraging grounds in no-take zones for dugongs from 17% to 42% and green turtles from 0.03% to 13% of the total area of occupancy identified for each species [10], [31], [32]. The areas for protection built on the pre-2003 management arrangements including the Dugong Protection Areas (DPAs) [33] and were predominantly identified using expert opinion and data from aerial surveys, seagrass mapping, and sighting records [31], [32], [34]. Such approaches may not satisfy stakeholders [35] who can better identify with information on individual animals.

Dugongs and green turtles are often found together, sometimes at high densities [26], and thus may be managed together (e.g., “Go-slow” areas in Moreton Bay near Brisbane, www.nprsr.qld.gov.au/parks/moreton-bay/about.html). However, a comprehensive understanding of the comparative ecology of dugongs and green turtles is required to optimize management because a comparative study on the stomach contents of dugongs and green turtles in Torres Strait found that dugongs fed exclusively on seagrasses whereas turtles consumed both seagrasses and macroalgae [36]. Thus a detailed and concurrent study of the movements of both species is required.

We used fast-acquisition GPS satellite telemetry to quantify the fine-scale distributions of sympatric dugongs and green turtles to enhance their management by tracking them at two locations of high conservation value in northeastern Australia (Shoalwater Bay and Torres Strait). Home-range analysis and bathymetric modeling were then used to compare spatial use within and between species and within and between geographic areas. Our objectives were to: (1) determine the spatial relationships between dugongs and green turtles in regions where they co-occur and between different locations; (2) assess the biological appropriateness of developing techniques to co-manage these two species; (3) evaluate the efficacy of the existing protection zones at our study locations. We also discuss how the management of dugongs and green sea turtles and other sympatric, mobile marine megafauna can be enhanced by detailed studies of the movements of individual animals.

## Materials and Methods

### Ethics Statement

All necessary permits were obtained for the described study. The animal use protocol for this research was approved by the James Cook University Animal Ethics Committee and Queensland Parks and Wildlife. All protected species were handled in strict accordance with local and international regulations. The dugong research was conducted under James Cook University Animal Ethics Committee Permits A1498 and A1683 and Queensland Parks and Wildlife Scientific Purpose Permit WISP006774410. The turtle research was conducted under James Cook University Animal Ethics Committee Permits A1229 and A1683 and Queensland Parks and Wildlife Scientific Purpose Permits WISP02742310 and WISP09469711.

### Satellite Transmitter Attachment and Tracking

Raw, unfiltered tracking data were collected using fast-acquisition GPS satellite transmitters attached to six dugongs (three females and three males) and four adult female green sea turtles near Mabuiag Island, Torres Strait, Australia (9° 57'S, 142° 10'E) in July 2009 and September 2010, and five dugongs (four females and one male) and six female green sea turtles (five adults and one prepubescent) in Shoalwater Bay, Australia (22° 25′S, 150° 25′E) in June/July 2012 (Table 1).

**Table 1:** Information on the dugongs and green sea turtles tagged in Shoalwater Bay, Australia in 2012 and Torres Strait, Australia in 2009 and 2010.

Individual	Date tagged	Sex	Maturity	Length (cm)*	Days+	Tag type
Shoalwater Bay						
Dugongs						
652631A	01 July 2012	M	Unknown	262	141	Telonics
652636A	30 June 2012	F	Adult	288	51	Telonics
652640A	01 July 2012	F	Unknown	252	6	Telonics
652642A	01 July 2012	F	Immature	179	34	Telonics
652643A	30 June 2012	F	Immature	229	54	Telonics
Turtles						
96777	01 July 2012	F	Adult	96.1	145	Sirtrack
96780	03 July 2012	F	Adult	97.3	121	Sirtrack
108469	29 June 2012	F	Adult	104.5	147	SMRU
108472	29 June 2012	F	Adult	100.5	148	SMRU
120640	30 June 2012	F	Prepubescent	102.1	137	Wildlife Computers
120641	01 July 2012	F	Adult	95.5	131	Wildlife Computers
						
Torres Strait						
Dugongs						
641060A	18 September 2010	F	Adult	250	69	Telonics
641058A	13 September 2010	F	Likely Adult	224	33	Telonics
641052A	22 September 2010	M	Large Juvenile	182	79	Telonics
641054A	22 September 2010	M	Likely Adult	224	30	Telonics
641057A	14 September 2010	F	Adult	335	22	Telonics
641055A	14 September 2010	M	Likely Adult	223	7	Telonics
Turtles						
70455	22 September 2010	F	Adult	118	134	Sirtrack
95889	25 July 2009	F	Adult	98	27	Sirtrack
95891	25 July 2009	F	Adult	102.1	28	Sirtrack
95892	21 September 2010	F	Adult	105.6	47	Sirtrack

^*^Dugong length was measured as straight total length [37], turtle length was measured as curved carapace length (CCL) [38].

+ Total days tracked while foraging.

The dugongs were captured using the dermal holdfast technique [39] in Torres Strait and the standard rodeo technique in Shoalwater Bay [40]. At both locations, the dugongs were fitted with Telonics Gen 4 GPS/ARGOS marine units attached to a 3 m tether linked to a padded tailstock harness (for details see [41]).

The green turtles were captured using the standard rodeo technique [42], brought to Mabuiag Island (Torres Strait) or MacDonald Point (Shoalwater Bay), and fitted with one of four types of satellite transmitters (Sirtrack F4G 291A, Wildlife Computers SPLASH10 BF-273A and Splash10 BF-273C, or SMRU SRDL 9000x). Each transmitter was attached to the carapace using the methods described in Shimada et al. (2012) [43]. Each turtle was released from shore the day after capture.

Dugong units were programmed to collect a GPS position hourly; turtle units every 30 minutes. All units were programmed with a five minute repeat in case a signal was not received when the animal surfaced.

### Data Processing

All raw data were transmitted via the ARGOS network, downloaded from ARGOS via the transmitter manufacturer supplied software, converted to the Universal Transverse Mercator (UTM) coordinate system, filtered, and processed.

Dugong location data were used from the time of release until a transmitter stopped transmitting or detached. Post-release locations were not removed from the dataset as previous studies indicated no behavioral changes after capture and handling [44]. Transmitter detachments were identified by: (1) acquisition of successive succeeded GPS location classes at consistent time intervals (e.g., on the hour, every hour), (2) prolonged straight line movement, or (3) signals from one location for an extended period of time (e.g., days).

The location data for turtles were not used in our analysis until the target animal returned to its capture location or exhibited foraging behavior. Foraging behavior was identified by non-directed movement (i.e., tortuous, short distance movements) for a minimum of three consecutive days after an initial directed movement (i.e., straight-line movement covering a significant distance over a short period of time) [45]. The tortuosity of individual movements was evaluated through visual inspection of connecting lines between consecutive location points using GIS. Straight-line movement was defined by the trajectory between two locations being less than 45° off to either side from the extension of the previous two locations [45].

Different filtering techniques were required for each type of transmitter because of differences between transmitters, locational accuracy, and species life-history characteristics. The Telonics units used for dugongs employed a Quick Fix Pseudoranging (QFP) alternative when a GPS location could not be acquired. QFP locations were categorized by locational accuracy into three categories: resolved QFP, resolved QFP (uncertain), and unresolved QFP. Telonics (2012) [46] states that 98.4% of resolved QFP positions are within 30 m of the actual position, resolved QFP (uncertain) positions are generally within 75 m, and unresolved QFP positions are within several hundred meters. Because the use of QFP technology in animal tracking is relatively new, objective filtering techniques were developed to maintain accuracy of acquired locations. For fine-scale analysis, data were initially filtered by location class, using only succeeded GPS, resolved QFP, and resolved QFP (uncertain) location classes to maintain accuracy ≤75 m. After initial filtering, over-speed errors were removed; these were identified by the distance and time between successive fixes necessitating speeds beyond the maximum sustained swimming speed for dugongs of 10 km hr^-1^ [47]. Locations more than 30 m inland were also removed as this is the error estimate for the most precise QFP locations.

For turtles, all units used fast-acquisition GPS technology and were filtered in two ways. First, following the manufacturer's instructions, initial filtering used the residual error and number of satellite uplinks used for each location. Locations with a residual error greater than 30 or with fewer than four satellite uplinks were excluded [48]. Second, data were filtered using the data-driven method described by Shimada et al. (2012) [43], which removes locations for which speeds between successive locations exceed the maximum linear speed (*V_max_*) of an animal or if all of the following apply: (a) the number of source satellites is four, (b) the inner angle is acute, and (c) the speed either from or to a subsequent location exceeds a maximum “loop-trip” speed (*V_lp_*) calculated for an animal [43]. *V_max_* for green sea turtles was determined to be 9.9 km hr^-1^ and *V_lp_* was 2.0 km hr^−1^ (for details on how *V_max_* and *V_lp_* were calculated see [43]). Our calculated *V_max_* for green turtles is consistent with maximum speeds calculated by Heithaus et al. (2002) [49] (median: 11 km hr^−1^; inter-quartile range: 10–12 km hr^−1^). Finally, locations were compared with Landsat imagery and digital elevation models to determine if locations found on land were accessible to a basking turtle and implausible locations were removed.

After filtering, the location data from each dugong or turtle were standardized by dividing the remaining location points into 3 hour duty-cycles and selecting the most accurate location within each duty-cycle. This duration was chosen to retain as many location points as possible while minimizing differences in the number of location points per day per animal. In addition, duty-cycles were used in an effort to reduce the effects of autocorrelation and effects resulting from differences in transmitter performance. These measures were necessary as sample size has been shown to significantly affect home-range estimates [50], [51]. The best location was chosen using two criteria: (1) the location with the greatest number of satellite uplinks, and (2) if multiple locations had the same number of uplinks, the final choice was the location with the lowest residual error (Sirtrack, Wildlife Computers, and SMRU units) or the lowest positional dilution of precision error (Telonics units). In the event multiple locations had the same error value, the location closest to the median time point within each duty-cycle was chosen.

### Home-range Estimation and Spatial Use

Home-ranges were calculated using fixed kernel density estimation with bandwidths selected by likelihood cross-validation (CVh) [52]. The fixed kernel method was selected over the adaptive kernel method because it has been shown to produce more accurate and precise home-range estimates that are less sensitive to autocorrelation [51], [53]. While least-squares cross-validation (LSCV) has been suggested as the best bandwidth selector by many studies [51], [54], we found that it produced elongated ranges that were skewed along a single axis; a by-product of the layout of the locational data of animals within this study and most likely a misrepresentation of each animal's actual use of space. After testing and interpreting the ecological/behavioral relevance of several other bandwidth selectors, CVh was found to produce ranges which were not skewed and the most ecologically appropriate. Hemson et al. (2005) [55] and Horne & Garton (2006) [56] also found that this method sometimes performs better than LSCV.

Kernel densities and bandwidths were calculated using the Geospatial Modelling Environment (GME), an extension to ArcGIS [57], [58], with a resolution of 50 m. This resolution was selected because the mean accuracy of filtered fast-acquisition GPS locations is within 50 m of the true location [43] and the majority of filtered Telonics locations in this study were classified as resolved QFP or higher (mean  = 83.5, SE  = 5.3%, n = 11). GME was also used to generate 95% and 50% contour polygons from the calculated kernel densities in vector format. 95% home-range and 50% core range areas were calculated in ArcMap 10 [59] from the 95% and 50% contour polygons. Regions of contour polygons that overlapped land were removed before final areas were calculated. A core area was defined as the area in which an individual is predicted to be 50% of the time.

Home-ranges were calculated for each animal using data from the entire period in which they were tracked. We plotted days tracked versus range size to determine if there was a relationship and found no significant correlation between days tracked and range size with the majority of animals showing range stabilization at or near 20 days of tracking (Figure S1; Table S1). Diurnal comparisons were made by calculating two home-ranges for each animal using all filtered data, recorded during the day (0600–1800 hours) or night (1800–0600 hours). Differences between day and night ranges were determined in two ways: (1) visual determination of differences in location of animal movements (e.g., distance from geographic features) and (2) differences in estimated range size. Combined home-ranges were calculated for each species by merging the 95% and 50% ranges of all individuals to determine the total area covered.

### Depth Zone Distribution

Bathymetry layers of each region were used to determine the depth zones used by each tracked animal. The Torres Strait layer was developed in 2008 at a resolution of 110 m [60] and the Shoalwater Bay layer was developed in 2010 at 100 m resolution [61]. Depths were measured at mean sea level and depth profiles were calculated by interpolation of data points from a variety of survey methods [60], [61]. Each layer was stored in raster format, reclassified into 5 m depth zones and converted to vector format to allow overlay operations with home-range vector polygons. Total individual ranges and the diurnal ranges of each individual (both 95% home-ranges and 50% core areas) were then overlaid on the reclassified bathymetry layer and the total area over each depth zone was calculated for each individual.

## Results

### Tracking Duration and Location Points

The dugongs were tracked for between 6 and 141 days (mean  = 47.8 days, SE  = 11.7 days, n = 11) and foraging female turtles were tracked between 27 and 148 days (mean  = 106.5 days, SE  = 16.1 days, n = 10) (Table 1). Unfiltered data points ranged between 100 and 2846 locations (median: 465 locations) for dugongs and 196 and 2471 locations (median: 521 locations) for turtles. After data filtering, dugong transmitters provided a per-dugong median of 193 locations (range: 41–965 locations) and turtle transmitters provided a per-turtle median of 210 locations (range: 118–732 locations) over the total tracking time for each individual. All transmitters provided roughly an equal number of locations during the day and night after data filtering (Table S2). One dugong from Shoalwater Bay (652642A – female) became stranded during tracking with the transmitter unit still attached. Location fixes and time-depth recorder (TDR) data suggested the dugong started drifting with the current after 33 tracking days. All location points after the approximate time of death were excluded from the analysis. Inspection of the dugong carcass and photos of the stranded animal did not reveal any apparent cause of death.

### Home-ranges and Core Areas - Shoalwater Bay

**Figure 1:**
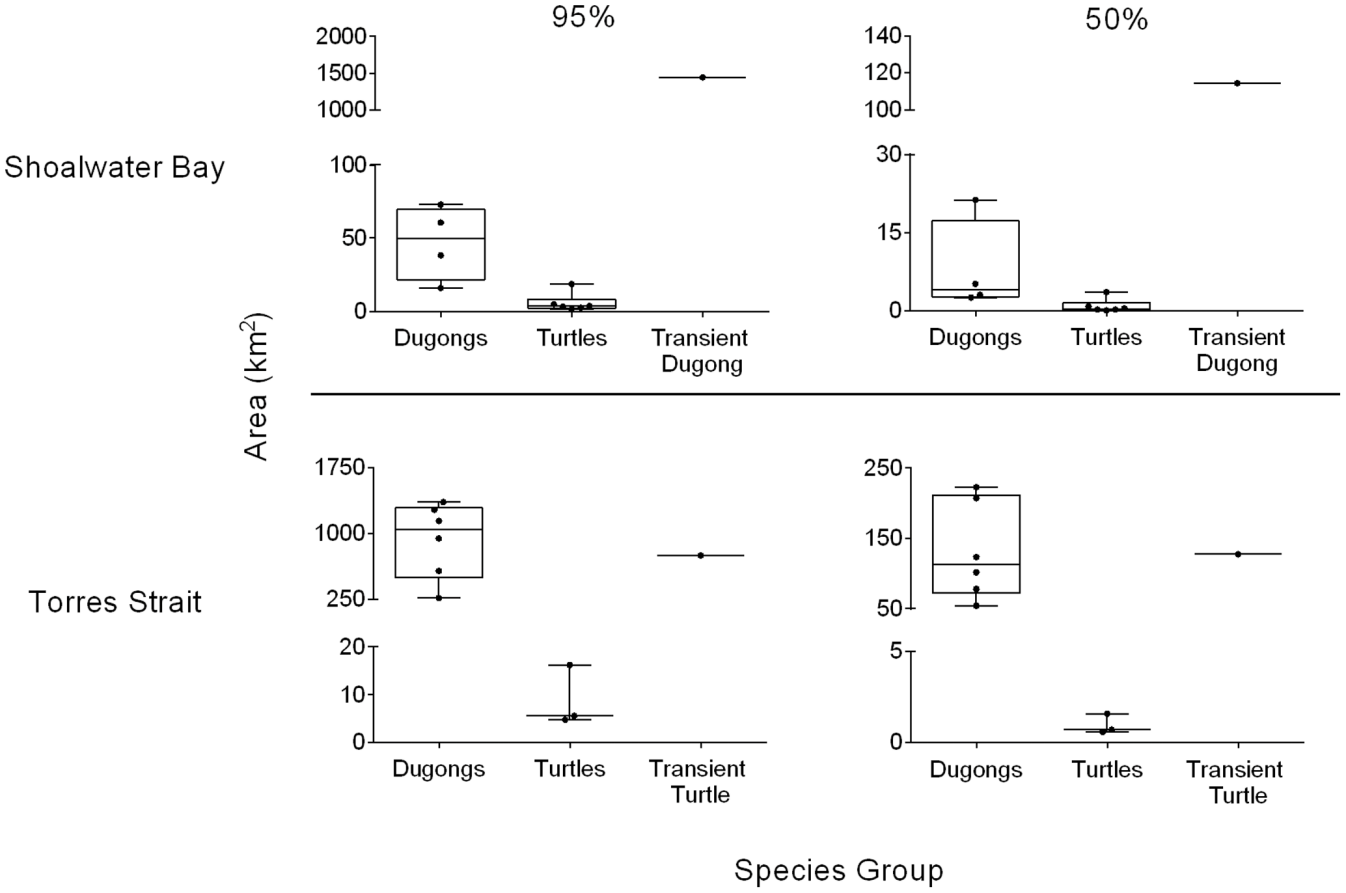
Home-range and core area size of tracked dugongs and green turtles. Comparison of 95% home-ranges (left) and 50% core areas (right) between Shoalwater Bay, Australia dugongs (n = 5), green sea turtles (n = 6) and a transient dugong (n = 1) (top) with Torres Strait, Australia dugongs (n = 6), reef associated green sea turtles (n = 3), and a transient green sea turtle (n = 1) (bottom) over the total tracking time of each animal. Lines within boxes represent the median, boxes represent interquartile range, whiskers represent minimum and maximum values, and dots indicate values for each individual. Note differences in scale on y axes.

Four of the five dugongs used relatively small ranges with 95% home-range areas ranging from 15.9 km^2^ to 72.8 km^2^ (median: 49.5 km^2^) and 50% core areas ranging between 2.6 km^2^ and 21.3 km^2^ (median: 4.2 km^2^), encompassing a total area of 123.7 km^2^ (95%) and 28.5 km^2^ (50%) (Figure 1; Table S2). The fifth individual (652636A – female) had a significantly larger range with a 95% home-range of 1444.6 km^2^ and 50% core area of 114.4 km^2^ and exhibited behavior consistent with a transient animal.

**Figure 2:**
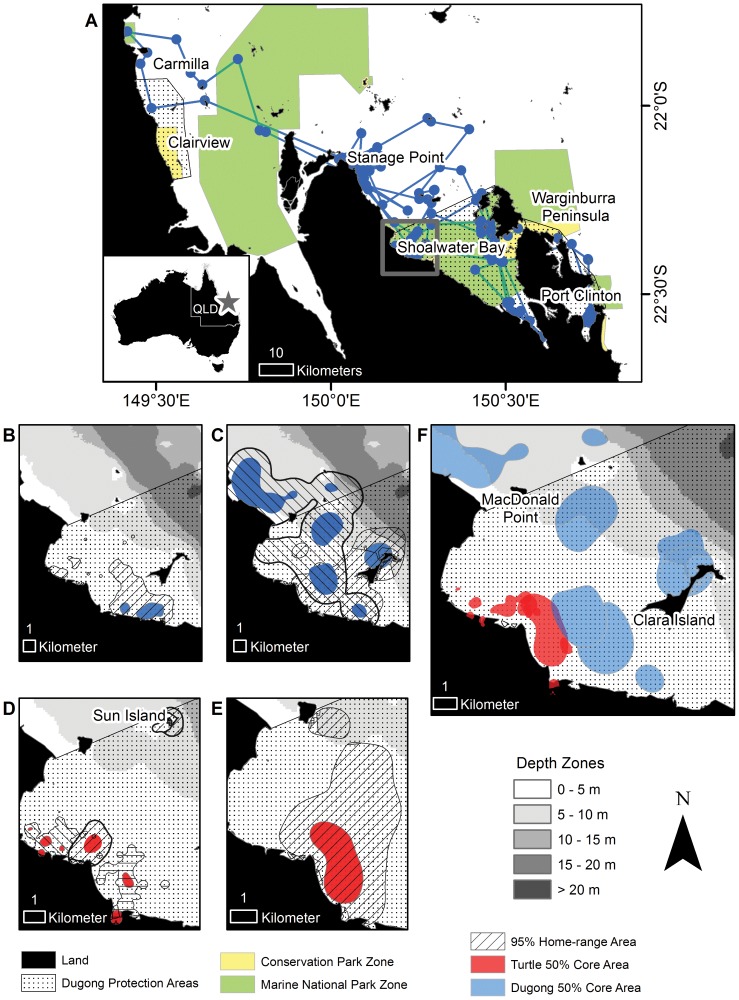
Shoalwater Bay dugong and green turtle spatial use. Dugong and green sea turtle tracking and foraging home-ranges within Shoalwater Bay, Australia using the total tracking duration of each animal. Blue features indicate dugongs and red features indicate green sea turtles. (**A**) Tracking locations and migratory path of the transient dugong (652636A). (**B** and **C**) Example home-ranges of dugongs (**B**: 652642A, **C**: 652640A (left) and 652643A (right)). (**D** and **E**) Example home-ranges of green sea turtles (**D**: 96780 (left), 108469 (middle), and 120640 (right); **E**: 120641). (**F**) Comparison of dugong and green sea turtle 50% core areas in northwestern Shoalwater Bay. Gray box indicates region displayed in maps **B-F**. For maps with multiple individuals plotted, each individual's 95% home-range is delineated by hatching at a different angle. Note differences in scale for each map.

All five dugongs were individualistic in their movements. The four dugongs with small 95% home-ranges (<75 km^2^) were resident within the northwest portion of Shoalwater Bay between Clara Island and MacDonald Point for most of the tracking period (Figure 2B,C). One of these dugongs (652631A – male, 141 days) relocated approximately 90 km to the west near Clairview on 26 September 2012 (after two days at large) and spent the remainder of its tracking time at this location.

Four dugongs used the existing Dugong Protection Area (DPA [restrictions of gill netting; [33]]) and Great Barrier Reef Marine Park (GBRMP) marine national park zone (green zone [all fishing banned]) within Shoalwater Bay. Two of these animals (652643A – female and 652642A- female) spent their entire tracked time within the DPA and green zone, one relocated (652631A – male) from the Shoalwater Bay DPA (some restrictions of gill netting [33]) and green zone directly to the Clairview Bluff – Carmilla Creek DPA and GBRMP conservation park zone (yellow zone), thus all of its presumed foraging range was within the boundaries of these zones. The fourth dugong (652636A – female) made several large-scale movements around the region, moving throughout Shoalwater Bay and making an exploratory loop-trip 90 km to the west to Carmilla and then back into Shoalwater Bay over three days (Figure 2A). This animal spent two days in Port Clinton, 75 km away along the eastern side of the Warginburra Peninsula, before returning to Shoalwater Bay. Despite making this larger scale movement, this dugong had a preferred core area located at the northwest edge of Shoalwater Bay near Stanage Point and most (60%) of its 95% home-range and core areas fell outside DPA and GBRMP protection zones.

Five of the six green turtles tracked in Shoalwater Bay had very small coastal ranges that were closer to shore than those of the dugongs (Figure 2D,E,F). The sixth turtle (120641) was much more mobile than the others and had a significantly larger range (95%: 18.6 km^2^, 50%: 3.6 km^2^). This animal showed space use similar to that of dugongs within the area (Figure 2F). Combined turtle 95% home-range areas ranged from 1.4 km^2^ to 18.6 km^2^ (median: 3.5 km^2^) and 50% core areas ranged between 0.1 km^2^ and 3.6 km^2^ (median: 0.4 km^2^), encompassing a total area of 25.5 km^2^ (95%) and 5.1 km^2^ (50%) (Figure 1; Table S2). All six individuals remained near the coast in the northwest portion of Shoalwater Bay between MacDonald Point and Clara Island, close to their points of capture. Each turtle displayed strong site fidelity and had its own distinct core areas with very little overlap between other individuals' core areas. Four individuals (96777, 96780, 108469, and 108472) used mangrove areas and travelled up coastal streams with all but one of these individuals (108469) having core areas within these habitats. Location points along the shoreline suggested that all the turtles basked on land. One individual (108469) spent 2.5 weeks near Sun Island before relocating to the coast south of MacDonald Point. All turtles stayed within the Shoalwater Bay DPA and green zone and only a single turtle (108469) had a range that crossed outside of the DPA boundary, but this only accounted for <5% of this individuals 95% home-range and was still within the GBRMP green zone boundary. For both the dugongs and the green turtles, spatial use did not significantly differ in relation to geographic areas or between species on a monthly basis (Figure S2).

### Home-ranges and Core Areas – Torres Strait

The six dugongs tracked in Torres Strait used 95% home-ranges ranging from 264.3 km^2^ to 1269.2 km^2^ (median: 1042.9 km^2^) and 50% core areas ranging between 54.3 km^2^ and 222.8 km^2^ (median: 112.6 km^2^), encompassing a total area of 3861.9 km^2^ (95%) and 640.9 km^2^ (50%) (Figure 1; Table S2). These ranges were significantly larger than four of the Shoalwater Bay dugongs, but not the transient dugong (652636A – female).

**Figure 3:**
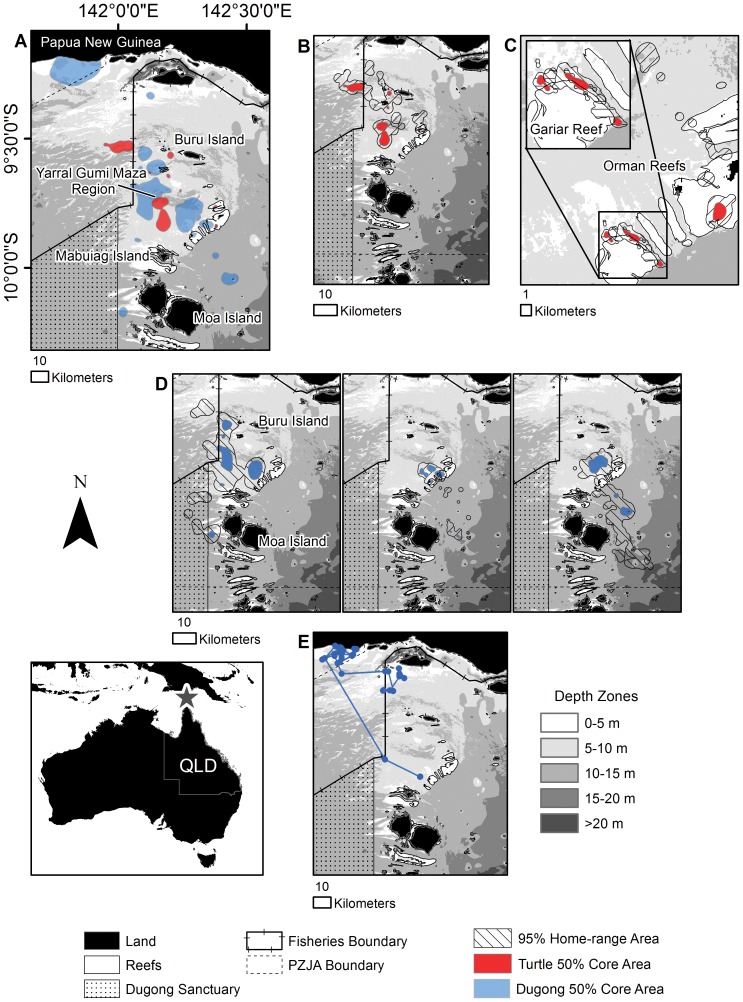
Torres Strait dugong and green turtle spatial use. Dugong and green sea turtle tracking and foraging home-ranges within Torres Strait, between the northern tip of Australia and Papua New Guinea using the total tracking duration of each animal. Blue features indicate dugongs and red features indicate green sea turtles. (**A**) Comparison of dugong and green sea turtle 50% core areas in Torres Strait. (**B**) Home-range of the transient turtle (95889). (**C**) Home-ranges of reef associated turtles (inset is a close up of Gariar Reef where two turtles were resident (70455 and 95892). (**D**) Example home-ranges of dugongs (left: 641058A, middle: 641054A, right: 641052A). (**E**) Tracking locations and migratory path of the dugong that traveled to Papua New Guinea (641055A). PZJA  =  Protected Zone Joint Authority. For maps with multiple individuals plotted, each individual's 95% home-range is delineated by hatching at a different angle. Note differences in scale for each map.

As in Shoalwater Bay, the movements of the Torres Strait dugongs were individualistic with multiple core areas. Most animals spent a proportion of their time at a foraging site between Mabuiag and Buru Islands known as the Yarral Gumi Maza region, which is generally over 5 meters deep (Figure 3A,D). Two animals (641052A – male and 611057A – female) made movements towards the southeast boundary of the Torres Strait Protected Zone Joint Authority (PZJA) region, one individual (641054A – male) spent the majority of its time around the Orman Reefs, another animal (641055A – male) moved north to the coast of Papua New Guinea (Figure 3E), and the remaining two dugongs (641060A – female and 641058A – female) remained within the Yarral Gumi Maza region. Only one dugong (641054A – male) ventured over the reef flat and lagoon of the Orman Reefs, but this animal spent the majority of its time away from reefs in deeper-water areas and relocated off the east coast of Moa Island on 16 October 2010 where it remained for the remainder of the time it was tracked (Figure 3D). A single dugong (641058A – female) crossed into the existing Dugong Sanctuary; however, 85% of its range was outside of the Sanctuary. One individual (641052A – male) made an exploratory loop-trip of 80 km south to the Cape York Peninsula and northern GBR over a period of two days. This individual did not enter the GBRMP.

Three Torres Strait turtles used space very similarly to the Shoalwater Bay turtles with 95% ranges between 4.8 km^2^ and 16.2 km^2^ (median: 5.6 km^2^) and 50% core areas between 0.6 and 1.6 km^2^ (median: 0.7 km^2^), encompassing a total area of 25.0 km^2^ (95%) and 2.9 km^2^ (50%) (Figure 1; Table S2). Similar to the Shoalwater Bay turtles, the Torres Strait turtles showed strong site fidelity; each individual had its own distinct core areas. However, unlike turtles in Shoalwater Bay, which preferred coastal areas, Torres Strait turtles were reef associated with three of four turtles inhabiting the Orman Reefs and two of these individuals (70455 and 95892) remained on the northern side of Gariar Reef over almost the entire tracking period. These turtles only made short excursions (<1 km) over the reef flat and into the lagoon, spending most of their time near the reef crest and reef slope (Figure 3C). A single turtle (95889) was classified as transient, having a 95% range of 749.1 km^2^ and 50% core area of 127.4 km^2^, similar to the ranges of the Torres Strait dugongs (Figure 1). The transient turtle (95889) also spent a significant portion of time within the Yarral Gumi Maza region and northwest portion of Torres Strait towards Papua New Guinea (Figure 3B). No turtles crossed into the Dugong Sanctuary. For both the dugongs and the green turtles, spatial use did not significantly differ in relation to geographic areas or between species on a monthly basis (Figure S3).

### Diurnal Comparisons

There was no overall trend in differences in the size of dugong 95% home-ranges and 50% core areas between day and night. Conversely, green turtles in Shoalwater Bay and reef associated turtles in Torres Strait had more restricted ranges during the night, with significant decreases of up to 76% in nighttime home-ranges and core areas (Table S2). Dugongs and turtles in both regions did not show major differences in the geographic locations of their home-ranges and core areas between day and night.

### Depth Zone Distributions - Shoalwater Bay

**Figure 4:**
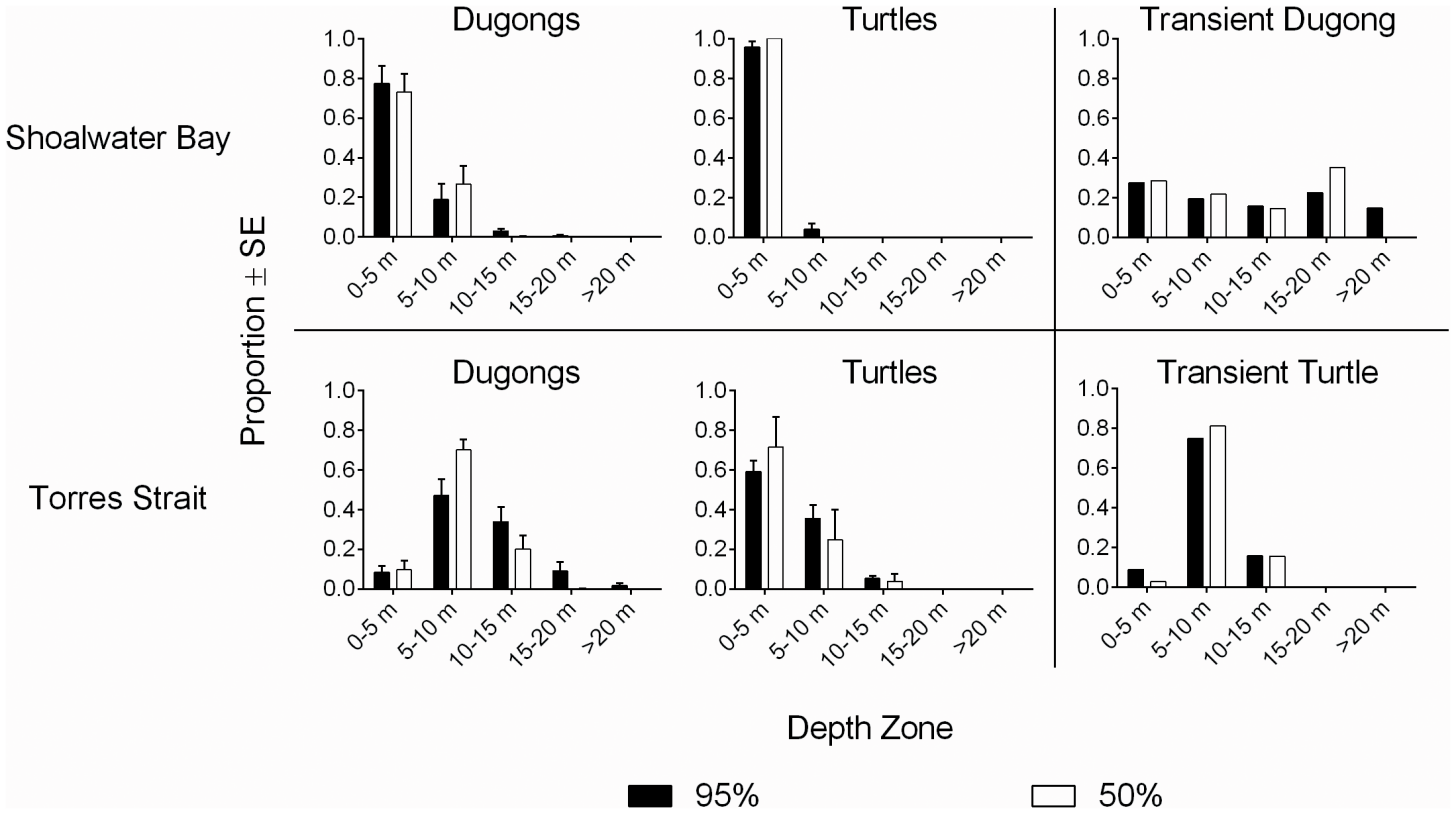
Depth zone use of tracked dugongs and green turtles. Depth comparison of 95% home-ranges and 50% core areas between Shoalwater Bay, Australia dugongs (n = 5), green sea turtles (n = 6), and a transient dugong (n = 1) (top) with Torres Strait, Australia dugongs (n = 6), reef associated green sea turtles (n = 3), and a transient green sea turtle (n = 1) (bottom).

Within Shoalwater Bay, the tracked dugongs and turtles frequented shallow water areas between 0–5 m (Figure 4). Turtles were found almost exclusively in intertidal areas with all core areas found in depth zones less than 5 m and over tidal flats, within mangrove zones, or up tidal creeks (Figure 2D,F). Only three turtles (108469, 108472, and 120641) had 95% home-ranges extending over regions >5 m in depth and these deeper areas accounted for only a small percentage of their ranges (mean  = 8.3%, SE  = 4.6%). No turtles were found in waters >10 m deep. Resident dugongs were found predominantly in subtidal zones with around 80% of their 95% and 50% ranges found in depth zones between 0–5 m (Figure 4). The remainder of the dugong ranges were in the 5–10 m zone; some individuals occasionally ventured over deeper areas up to a maximum of 20 m in depth. The transient dugong (652636A – female) was found over a range of depth zones between 0–20 m, with roughly equal portions of its range found over each 5 m depth zone (Figure 4). None of the dugongs had core areas over the tidal flats preferred by turtles (Figure 2F). There were no diurnal differences in the depth preferences of dugongs or turtles in Shoalwater Bay.

### Depth Zone Distributions - Torres Strait

Torres Strait dugongs and the transient turtle had similar distributions over depth zones. Animals were found mostly over deeper-water areas with depths of 5-15 m. Core areas were centered around the 5-10 m depth zone with 75% of each individual's range lying within these depths. Over half of the 95% home-range of two dugongs (641057A – female and 641052A – male) were over depths >10 m, with a maximum depth of 25 m. Reef associated turtles showed preference for regions with depths between 0–10 m, with >90% of their 95% ranges over these depths (Figure 4). All Torres Strait animals displayed significantly different depth zone preferences to animals in Shoalwater Bay with dugongs and turtles found over deeper depth zones in Torres Strait. Diurnal comparisons showed no differences in the depth preferences of dugongs, reef associated turtles, or the transient turtle.

## Discussion

This study is the first to simultaneously track dugongs and green sea turtles within their foraging grounds to compare the habitat use of these two sympatric megaherbivores. Fast-acquisition GPS tracking of dugongs and green turtles confirmed that they use similar areas in two separate regions where they co-occur with a significant amount of overlap in their ranges, despite most of the turtles using much smaller areas than the dugongs. However, at both locations some turtles showed patterns of space use similar to the dugongs. These results suggest that spatial management could be systematically designed to co-manage these species using fine-scale satellite tracking to identify key areas of habitat use to supplement survey data. Satellite tracking also showed that management plans may need to be designed to accommodate specific differences in range sizes and usage of different depth zones specific to each region.

### Spatial Relationships and Regional Comparisons

Dugong range sizes and depth zone preferences differed between localities with dugong ranges in Torres Strait being much larger than those in Shoalwater Bay and other regions where they have been tracked (e.g., Hervey Bay and Cleveland Bay, Australia; Lease Islands, Indonesia), by several orders of magnitude (average range from other regions: 24.0–63.6 km^2^; this study: Torres Strait; median  = 942.6 km^2^, Shoalwater Bay; median  = 60.6 km^2^) [41], [62], [63]. Dugongs in Torres Strait also preferred deeper-water habitats (>5 m), rather than the tidal and shallow sub-tidal habitats observed in Shoalwater Bay (this study) and other areas (e.g., Moreton Bay, Shark Bay, and Hervey Bay, Australia) [64], [65].

In contrast, most of the green sea turtles (n = 8) we tracked used small, restricted ranges in relatively shallow water, with high site fidelity, and limited nighttime ranges; a result similar to green turtles in other regions [66]–[68]. However, the presence of green turtles displaying behavior similar to dugongs (n = 2) in both regions suggests that some green turtles may be influenced by environmental factors and geographic features in a similar way to dugongs. These differences and similarities between species and regions may be associated with the relative availability of deeper-water and shallow-water habitat, the presence of reefs, the location and size of available seagrass meadows, and the regional dugong and turtle population sizes.

Torres Strait provides considerable areas of deeper-water habitat between 5 and 15 m in depth with shallow-water areas accounting for only a small percentage of available area which is primarily restricted to reef-top zones [60]. Additionally, most seagrass in Torres Strait is found within these deeper-water zones, which support an estimated 13,425 km^2^ to 17,500 km^2^ of seagrass habitat, the largest continuous seagrass area in Australia [69]–[71]. Reef-top areas also support productive seagrass pastures, but at a significantly smaller scale (e.g., ∼95 km^2^ at the Orman Reefs) [72], [73]. Dugong access to reef-top seagrass meadows is limited by tides [74]–[76] whereas the deeper-water seagrass is continuously available.

In contrast, along the coastline of eastern Australia south of Cooktown, which includes Shoalwater Bay, most seagrass meadows are much smaller (range: 55–1843 km^2^), primarily restricted to coastal tidal and sub-tidal flats [77] in shallow, sheltered bays, and thus fragmented into multiple, disjunct pastures, creating a much smaller area of available habitat for dugongs and turtles. Shoalwater Bay supports the largest area of seagrass (130 km^2^) in the southern GBR [77], [78]. This pattern of seagrass distribution may explain the smaller range sizes of the dugongs tracked in Shoalwater Bay and in other studies [41] along with the shallower depths used by both species.

### Spatial Zoning and Regional Management

The regional differences in the spatial patterns of dugongs and green sea turtles within Torres Strait and Shoalwater Bay have significant implications for the management and conservation of these two species. In Torres Strait, dugongs and green sea turtles cross jurisdictional boundaries, and they cross these boundaries in all directions. This situation requires management to be co-developed with neighboring regions to enable protection throughout each species' range. This approach should be used to extend the community based management planning, currently developed only at the scale of the sea-country of individual communities [29]. In contrast, dugongs and green turtles along the GBR have much more restricted ranges and do not cross international boundaries while on their feeding grounds. Thus, coordinated management decisions at state and federal levels are appropriate for most of the life cycles of both species. However, because some GBR green turtles cross international boundaries during breeding migrations [79] and dugongs occasionally make individualistic large-scale movements to foraging grounds outside the GBR [41], management plans also need to incorporate the likelihood of cross-jurisdictional movements.

The overlap in the home-ranges of dugongs and green turtles shows that protection measures, such as MPAs, no-take reserves, and seasonal spatial closures, could be developed for either species and, by default, spatial protection would encompass at least a portion of the range of the other. However, focusing conservation efforts on the space use of both species in a coordinated management scheme may be a more cost-effective approach as dugongs and green turtles appear to have different preferences for core areas and depth zones which need to be taken into account when designating protection zones. Also, co-management of dugongs and green turtles may act as an umbrella for many other species that share these habitats within the GBR/Queensland coast and Torres Strait.

Under current Australian national legislation, green turtles are listed as threatened species, while dugongs are not. Because of this, the triggers for significant impact are easier to meet for green turtles than they are for dugongs. This difference enables dugong management to be triggered by green turtle management. Alternatively, the broad-scale spatial distribution of dugongs is much better known than for green turtles [80] as dugongs being larger are more reliably detected using aerial-surveys than turtles, which are also difficult to identify species from the air. Thus, marine spatial management in Australia is generally better developed for dugongs than it is for green turtles. Through complimentary co-management of both species aided by satellite tracking of individual animals, dugong spatial management could be used to designate regions for protection that are important to both species, while green turtles may act as a catalyst for setting other conservation and environmental management plans into action.

### Efficacy of Existing Protection Zones

The efficacy of current protection zones also appears to differ among regions, although our conclusion is tentative because of our small sample sizes of tracked animals. The existing Torres Strait Dugong Sanctuary was only used by a single tracked dugong and accounted for less than 15% of this individual's total range. This lack of spatial use of the Dugong Sanctuary by our tracked turtles and dugongs _ENREF_57supports conclusions based on extensive aerial surveys that the Sanctuary should be extended [29]. Spatial closures for dugongs and green turtles in the Torres Strait region would be more effective if the current Dugong Sanctuary also protected green turtles and sanctuary areas were extended; negotiations are currently progressing to this end.

In contrast, existing protection areas in Shoalwater Bay appear to be adequate for the protection of dugongs and turtles within the region, with all tracked turtles and the majority of dugongs found within protection zone boundaries. However, the effectiveness of these reserves is unknown if the geographic distribution of food resources changes due to environmental and anthropogenic factors or if local dugong and turtle populations increase. Adaptive management of the region in addition to combined management of terrestrial and marine systems will be required to maintain the effectiveness of these protected areas, especially as the human population increases in Australia. Additionally, identification of migratory corridors, dugong breeding grounds, and source/sink habitats will further increase the efficacy of protection.

More broadly, these results show that fine-scale tracking of individual animals can provide important information on where animals travel and where their preferred habitats lie. This information can then aid in the delineation of MPA boundaries and assess the efficacy of existing MPAs that were created using other approaches such as density surveys, habitat mapping, and expert opinion.

## Conclusions

We have demonstrated the potential of fine-scale satellite tracking for improving species co-management, increasing the efficacy of existing MPAs and other protected zones, and identifying new areas for protection. Our results are preliminary and cannot be applied at the population level as sample sizes for both species were low, only two turtles classified as transient were tracked, and animals were only tracked during a single season in each location. Distribution patterns may be markedly different between seasons and/or years, especially in relation to changes in seagrass and algae distributions. Continued monitoring and larger sample sizes are required to further inform this evidence-based approach. It should also be noted that range sizes for individuals with tracking durations less than 20 days may be slightly larger or smaller than what we have shown due to fluctuations in range size correlated with tracking durations. However, the general areas they inhabit can be captured with short tracking periods providing useful information for comparisons between species. The findings of the present study have broad implications for the management of other sympatric species, mobile species dependent on benthic environments of variable quality, and predator-prey relationships. Examples of other co-occurring species that may benefit from this approach include manatees and green turtles; tiger sharks, dugongs, and sea turtles; and dolphin species which form mixed-species assemblages.

## Supporting Information

Table S1 **Evaluation of home range size and days tracked for each individual with a tracking duration greater than 20 days.**
(DOCX)

Table S2 **Total location points, filtered location points, and range sizes for each individual.**
(DOCX)

Figure S1 **Range size versus tracking duration for all tracked individuals with tracking durations greater than 20 days.**
(DOCX)

Figure S2 **Home-ranges and core areas of Shoalwater Bay, Australia dugongs and green sea turtles plotted by month.**
(DOCX)

Figure S3 **Home-ranges and core areas of Torres Strait, Australia dugongs and green sea turtles plotted by month.**
(DOCX)
