# Supplementary material for: Satellite Tracking of Sympatric Marine Megafauna Can Inform the Biological Basis for Species Co-Management
Source: PLoS One. 2014 Jun 3;9(6):e98944. doi: 10.1371/journal.pone.0098944 (PMC4043907; doi:10.1371/journal.pone.0098944)
Supplement: Table S1 — Evaluation of home range size and days tracked for each individual with a tracking duration greater than 20 days. (DOCX) [file pone.0098944.s001.docx]

| **Table S1.** Evaluation of home range size and days tracked for each individual with a tracking duration greater than 20 days using Pearson's correlation. | | | | | | | | |
| --- | --- | --- | --- | --- | --- | --- | --- | --- |
| **Individual** | **95% Home-range** | | | | **50% Core area** | | | |
|  | **r** | **t** | ***p*** | **df** | **r** | **t** | ***p*** | **df** |
| **Shoalwater Bay** |  |  |  |  |  |  |  |  |
| Dugongs |  |  |  |  |  |  |  |  |
| 652631A | -0.33 | -0.99 | 0.35 | 8 | -0.02 | -0.05 | 0.96 | 8 |
| 652636A | 0.57 | 1.39 | 0.24 | 4 | 0.03 | 0.06 | 0.95 | 4 |
| 652640A | Short tracking duration | | | | Short tracking duration | | | |
| 652642A | -0.99 | -9.29 | *p* < 0.05 | 2 | -0.99 | -9.31 | *p* < 0.05 | 2 |
| 652643A | -0.91 | -3.71 | *p* < 0.05 | 3 | 0.16 | -0.28 | 0.8 | 3 |
| Turtles |  |  |  |  |  |  |  |  |
| 96777 | -0.66 | -2.13 | 0.08 | 6 | -0.91 | -5.23 | *p* < 0.01 | 6 |
| 96780 | 0.76 | 2.82 | *p* < 0.05 | 6 | -0.26 | -0.65 | 0.54 | 6 |
| 108469 | -0.3 | -0.78 | 0.47 | 6 | -0.34 | -0.88 | 0.41 | 6 |
| 108472 | -0.13 | -0.32 | 0.76 | 6 | 0.06 | 0.16 | 0.88 | 6 |
| 120640 | 0.2 | 0.51 | 0.63 | 6 | -0.54 | -1.57 | 0.17 | 6 |
| 120641 | 0.74 | 2.73 | *p* < 0.05 | 6 | 0.38 | 1.02 | 0.35 | 6 |
|  |  |  |  |  |  |  |  |  |
| **Torres Strait** |  |  |  |  |  |  |  |  |
| Dugongs |  |  |  |  |  |  |  |  |
| 641060A | -0.19 | -0.34 | 0.76 | 3 | 0.69 | 1.67 | 0.19 | 3 |
| 641058A | 0.25 | 0.36 | 0.75 | 2 | 0.97 | 5.73 | *p* < 0.05 | 2 |
| 641052A | 0.87 | 4.26 | *p* < 0.01 | 6 | 0.29 | 0.73 | 0.49 | 6 |
| 641054A | Short tracking duration | | | | Short tracking duration | | | |
| 641057A | -1 | -77.58 | *p* < 0.01 | 1 | 0.93 | 2.6 | 0.23 | 1 |
| 641055A | Short tracking duration | | | | Short tracking duration | | | |
| Turtles |  |  |  |  |  |  |  |  |
| 70455 | -0.6 | -1.84 | 0.12 | 6 | -0.6 | -1.86 | 0.11 | 6 |
| 95889 | 1 | 20.74 | *p* < 0.05 | 1 | 1 | 21.98 | *p* < 0.05 | 1 |
| 95891 | -1 | -19.79 | *p* < 0.05 | 1 | -1 | -12.57 | 0.05 | 1 |
| 95892 | -0.8 | -2.33 | 0.1 | 3 | -0.84 | -2.67 | 0.08 | 3 |
|  |  |  |  |  |  |  |  |  |
