# Supplementary material for: Satellite Tracking of Sympatric Marine Megafauna Can Inform the Biological Basis for Species Co-Management
Source: PLoS One. 2014 Jun 3;9(6):e98944. doi: 10.1371/journal.pone.0098944 (PMC4043907; doi:10.1371/journal.pone.0098944)
Supplement: Table S2 — Total location points, filtered location points, and range sizes for each individual. (DOCX) [file pone.0098944.s002.docx]

**Table S2. Total location points, filtered location points, and range sizes for each individual.**

| **Individual** | **Total points** | **Filtered points** | **Filtered (Day)** | **Filtered (Night)** | **95%* (km^2^)** | **50%^+^ (km^2^)** | **95%* (km^2^) (Day)** | **50%^+^ (km^2^) (Day)** | **95%* (km^2^) (Night)** | **50%^+^ (km^2^) (Night)** |
| --- | --- | --- | --- | --- | --- | --- | --- | --- | --- | --- |
| **Shoalwater Bay** |  |  |  |  |  |  |  |  |  |  |
| Dugongs |  |  |  |  |  |  |  |  |  |  |
| 652631A | 2846 | 965 | 471 | 494 | 60.6 | 5.2 | 84.1 | 161.1 | 9.4 | 26.5 |
| 652636A | 465 | 220 | 82 | 138 | 1444.6 | 114.4 | 2207.6 | 1685.3 | 315.5 | 133.8 |
| 652640A | 130 | 44 | 22 | 22 | 72.8 | 21.3 | 72.5 | 88.2 | 22.5 | 26.2 |
| 652642A | 827 | 265 | 132 | 133 | 15.9 | 2.6 | 16.1 | 20.0 | 3.0 | 3.6 |
| 652643A | 1313 | 421 | 210 | 211 | 38.3 | 3.1 | 40.8 | 36.7 | 3.2 | 4.0 |
| Turtles |  |  |  |  |  |  |  |  |  |  |
| 96777 | 1163 | 580 | 288 | 292 | 1.4 | 0.1 | 1.4 | 0.9 | 0.1 | 0.1 |
| 96780 | 2173 | 732 | 367 | 365 | 2.5 | 0.3 | 3.0 | 1.5 | 0.6 | 0.2 |
| 108469 | 196 | 163 | 75 | 88 | 3.7 | 0.5 | 5.3 | 1.8 | 0.9 | 0.9 |
| 108472 | 687 | 500 | 248 | 252 | 4.9 | 0.9 | 5.8 | 4.5 | 0.9 | 0.9 |
| 120640 | 354 | 154 | 32 | 122 | 3.2 | 0.3 | 5.3 | 3.4 | 1.1 | 0.3 |
| 120641 | 264 | 210 | 87 | 123 | 18.6 | 3.6 | 26.0 | 14.0 | 4.9 | 3.2 |
|  |  |  |  |  |  |  |  |  |  |  |
| **Torres Strait** |  |  |  |  |  |  |  |  |  |  |
| Dugongs |  |  |  |  |  |  |  |  |  |  |
| 641060A | 425 | 160 | 73 | 87 | 572.9 | 101.7 | 559.1 | 448.0 | 122.3 | 80.1 |
| 641058A | 260 | 110 | 35 | 75 | 1269.2 | 222.8 | 1115.7 | 965.1 | 228.1 | 191.7 |
| 641052A | 1093 | 404 | 196 | 208 | 942.6 | 123.5 | 1868.4 | 1646.0 | 312.4 | 304.7 |
| 641054A | 1367 | 193 | 93 | 100 | 264.3 | 54.3 | 399.5 | 423.9 | 59.1 | 84.3 |
| 641057A | 239 | 125 | 53 | 72 | 1143.2 | 78.2 | 1207.3 | 1500.5 | 144.7 | 103.4 |
| 641055A | 100 | 41 | 17 | 24 | 1357.9 | 207.2 | 1107.3 | 2032.8 | 233.7 | 356.2 |
| Turtles |  |  |  |  |  |  |  |  |  |  |
| 70455 | 2471 | 394 | 203 | 191 | 5.6 | 0.7 | 6.7 | 3.8 | 1.1 | 0.6 |
| 95889 | 211 | 124 | 62 | 62 | 749.1 | 127.4 | 743.1 | 889.4 | 132.4 | 180.6 |
| 95891 | 206 | 118 | 70 | 48 | 16.2 | 1.6 | 21.4 | 18.0 | 2.3 | 2.3 |
| 95892 | 836 | 210 | 104 | 106 | 4.8 | 0.6 | 6.8 | 3.1 | 1.1 | 0.3 |
| *95% home-range area  ^+^50% core area | | | | | | | | | | |
