# Supplementary material for: Satellite Tracking of Sympatric Marine Megafauna Can Inform the Biological Basis for Species Co-Management
Source: PLoS One. 2014 Jun 3;9(6):e98944. doi: 10.1371/journal.pone.0098944 (PMC4043907; doi:10.1371/journal.pone.0098944)
Supplement: Figure S1 — Range size versus tracking duration for all tracked individuals with tracking durations greater than 20 days. (DOCX) [file pone.0098944.s003.docx]

**Figure S1.** Range size versus tracking duration for all tracked individuals with tracking durations greater than 20 days. Dashed line indicates approximate time of range stabilization for each species. Note differences in y axes.
