# Supplementary material for: Satellite Tracking of Sympatric Marine Megafauna Can Inform the Biological Basis for Species Co-Management
Source: PLoS One. 2014 Jun 3;9(6):e98944. doi: 10.1371/journal.pone.0098944 (PMC4043907; doi:10.1371/journal.pone.0098944)
Supplement: Figure S2 — Home-ranges and core areas of Shoalwater Bay, Australia dugongs and green sea turtles plotted by month. (DOCX) [file pone.0098944.s004.docx]

**Figure S2.** Home-ranges and core areas of Shoalwater Bay, Australia dugongs and green sea turtles plotted by month. July: all tracked dugongs (n=5) and turtles (n=6); August: all turtles (n=6), three dugongs (652631, 652636, 652643); September: all turtles (n=6), no dugongs; October: all turtles (n=6), no dugongs; November: five turtles (96777, 108469, 108472, 120640, 120641), no dugongs.
