# Supplementary material for: Satellite Tracking of Sympatric Marine Megafauna Can Inform the Biological Basis for Species Co-Management
Source: PLoS One. 2014 Jun 3;9(6):e98944. doi: 10.1371/journal.pone.0098944 (PMC4043907; doi:10.1371/journal.pone.0098944)
Supplement: Figure S3 — Home-ranges and core areas of Torres Strait, Australia dugongs and green sea turtles plotted by month. (DOCX) [file pone.0098944.s005.docx]

**Figure S3.** Home-ranges and core areas of Torres Strait, Australia dugongs and green sea turtles plotted by month. August: two turtles (95889, 95891); no dugongs; September: no turtles, all dugongs (n=6); October: two turtles (70455, 95892); five dugongs (641052A, 641054A, 641057A, 641058A, 641060A); November: one turtle (70455), two dugongs (641052A, 641060A).
